# Clinical significance of sirtuin 1 level in sepsis: correlation with disease risk, severity, and mortality risk

**DOI:** 10.1590/1414-431X202010271

**Published:** 2020-11-27

**Authors:** Xin Cheng, Senbing Zhang, Ye Wen, Zhihua Shi

**Affiliations:** 1 Department of Gynaecology, Xianning Central Hospital, The First Affiliated Hospital of Hubei University of Science and Technology, Xianning, China; 2 Department of Anesthesiology, Xianning Central Hospital, The First Affiliated Hospital of Hubei University of Science and Technology, Xianning, China; 3 Emergency Department, Xianning Central Hospital, The First Affiliated Hospital of Hubei University of Science and Technology, Xianning, China; 4 Hand and Foot Surgery, Xianning Central Hospital, The First Affiliated Hospital of Hubei University of Science and Technology, Xianning, China

**Keywords:** Sepsis, Sirtuin 1, Inflammation, Disease severity, Prognosis

## Abstract

This study aimed to investigate the value of sirtuin 1 (SIRT1) in differentiating sepsis patients from healthy controls (HCs), and its correlation with inflammation, disease severity, as well as prognosis in sepsis patients. Serum samples were collected from 180 sepsis patients and 180 age- and gender-matched HCs. The SIRT1 level in the serum samples was detected by enzyme-linked immunoassay. The clinical data of the sepsis patients were documented, and their disease severity scores and 28-day mortality rate were assessed. SIRT1 was decreased in sepsis patients compared with HCs, and the receiver operating characteristic curve (ROC) showed that SIRT1 distinguished sepsis patients from HCs (area under the curve (AUC): 0.901; 95% confidence interval (CI): 0.868-0.934). In sepsis patients, SIRT1 negatively correlated with serum creatinine (Scr), white blood cells (WBC), C-reactive protein (CRP), acute physiology, and chronic health evaluation II (APACHE II) score, and sequential organ failure assessment (SOFA) score, while it positively correlated with albumin. No correlation of SIRT1 with primary infection site or primary organism was observed. Furthermore, SIRT1 was reduced in 28-day non-survivors compared with 28-day survivors, and subsequent ROC showed that SIRT1 predicted 28-day mortality of sepsis patients (AUC: 0.725; 95% CI: 0.651-0.800), and its prognostic value was not inferior to Scr, albumin, WBC, and CRP, but was less than SOFA score and APACHE II score. In conclusion, measurement of serum SIRT1 might assist with the optimization of disease assessment, management strategies, and survival surveillance in sepsis patients.

## Introduction

Sepsis, the commonest cause of emergency admission to the intensive care unit (ICU) globally, develops as an overwhelming, systemic deregulated host response to a microbial infection, which leads to acute tissue damage and organ dysfunction, being associated with a high risk of mortality ([Bibr B01] [Bibr B02] 13). It is estimated that sepsis affected 48.9 million individuals worldwide in 2017 and it represents one of the leading causes of mortality (1). Pathologically, tissue hypoxia, mitochondria dysfunction, and cell apoptosis are important contributors of sepsis-induced organ dysfunction (4). Sepsis survivors suffer from mental and physical impairment and decreased quality of life (5). To date, the primary management of sepsis patients includes antibiotics and hemodynamic interventions (3). In spite of intensive clinical and investigational focus on the process of care and treatment of sepsis, the prognosis of sepsis patients remains poor due to the lack of effective biomarkers, and less-than-optimal treatment and supportive care (3). The investigation of effective biomarkers to facilitate earlier identification and intervention to improve prognosis in sepsis patients is of paramount importance.

Sirtuins (SIRTs), a highly conserved family of proteins first described in yeast, are composed of seven SIRTs (SIRT1-7) that are dispersed throughout different cell compartments, and are key regulators of inflammatory stress response in immune and non-immune cells via sensing NAD^+^ and deacetylating nearby histones (6). SIRT1 is the most extensively studied sirtuin, and it is reported to have a protective effect in inflammation-related multiple organ dysfunction of sepsis. Its main mechanism involves attenuating inflammatory response, improving antioxidant capacity, and reducing cell apoptosis (6-9). As an example, in a ligation and puncture murine model of sepsis, SIRT1 attenuates sepsis-induced lung inflammatory injury via repressing the inflammasome activation pathway and decreasing the production of proinflammatory mediators (8). Another study reveals that overexpression of SIRT1 facilitates proliferation but suppresses apoptosis of renal epithelial HK-2 cells via reducing the activation of nucleotide-binding oligomerization domain-like receptors inflammasome, which ameliorates lipopolysaccharide-induced acute kidney injury in sepsis (9). Although the mechanisms of SIRT1 underlying sepsis have been increasingly recognized, its clinical implication in sepsis patients remains to be elucidated. Herein, the aim of the present study was to investigate the potential of SIRT1 as a biomarker for disease management and prognosis prediction in sepsis patients.

## Material and Methods

### Subjects

Between January 2017 and September 2019, 180 sepsis patients admitted to our hospital (The First Affiliated Hospital of Hubei University of Science and Technology) were consecutively recruited for this study. All eligible patients were required to meet the following inclusion criteria: 1) confirmed diagnosis of sepsis in accordance with Surviving Sepsis Campaign: International Guidelines for Management of Severe Sepsis and Septic Shock, 2012 (10); and 2) older than 18 years. Patients were excluded from the study if they were pregnant or lactating women, or had known hematological disorders (e.g., leukemia, myelodysplastic syndrome, neoplastic metastases to bone marrow), other life-threatening diseases (e.g., malignant solid tumors), acquired immunodeficiency syndrome, or received immunosuppressive therapy for solid organ transplantation within 3 months before enrollment. Moreover, the current study also screened 180 healthy controls (HCs) from subjects who underwent health examination in this hospital from June 2019 to December 2019. HCs were age- and gender-matched with enrolled sepsis patients and had no history of severe infection or malignancies or other obvious abnormalities. This study was approved by the Ethics Committee of our hospital and all participants or their families provided written informed consents.

### Samples and clinical data collection

Blood samples of sepsis patients were collected within the first 24 h of ICU admission and before antibiotic therapy, which were divided into two parts: one was used for bacteria culture and necessary biochemical tests, and the other was used for the present study. Blood samples of HCs were collected when they were undergoing the health examination. After collection, samples were allowed to clot for 30 min at room temperature followed by centrifugation at 1200 *g* for 5 min at 4°C. The serum was removed from the clot, collected in cryogenic vials, labeled accordingly, and then stored at -80°C until subsequent detection. In addition, sepsis patients' clinical data were documented, including age, gender, body mass index (BMI), smoking status, chronic comorbidities [e.g., chronic obstructive pulmonary disease (COPD), cardiomyopathy, chronic kidney failure, and cirrhosis], biochemical indexes [serum creatinine (Scr), albumin, white blood cells (WBC), and C-reactive protein (CRP)].

### Disease severity assessments

For disease severity assessment, Acute Physiology and Chronic Health Evaluation (APACHE) II score and Sequential Organ Failure Assessment (SOFA) score were assessed within 24 h after admission (11). The APACHE II score system is composed of three parts, including physiologic scores ranging from 0 to 60 points, age scores ranging from 0 to 6 points, and comorbid disease scores ranging from 0 to 5 points, with a distribution of 0 to 71 points. A higher APACHE II score indicated a worse disease status. The SOFA scoring scheme assigned 1 to 4 points to each of the levels of dysfunction of six organs (respiratory, circulatory, renal, hematological, hepatic, and central nervous systems), with a total score of 0 to 24 points. A higher SOFA score was associated with more severe organ failure status.

### SIRT1 determination in serum samples

The SIRT1 level in serum of sepsis patients and HCs was detected by enzyme-linked immunoassay (ELISA) using human SIRT1 ELISA Kit (Shanghai Enzyme-linked Biotechnology, China), according to the manufacturer's instructions. Briefly, procedures of ELISA were performed as follows: firstly, the samples were diluted with diluent in 1:1 ratio, then 50 μL of diluted samples and 50 μL of standards were added into the reaction well. Next, 50 μL biotin-labeled antibody was immediately added into the well, followed by covering with film plate, gently mixing, and incubation at 37°C for 1 h. After washing and drying with absorbent paper, 80 μL of affinity chain enzyme (HRP) was added to each well, followed by gently mixing and incubation at 37°C for 30 min. After washing and drying with absorbent paper, 50 μL of chromogenic agent was added to each well, followed by gently mixing and incubation at 37°C for 10 min in the dark. Thereafter, 50 μL termination solution was added to each well, and the absorbance value of each well was immediately measured at 450 nm wavelength using a microplate reader (BD, USA). Finally, the SIRT1 level was calculated according to the standard curve.

### 28-day mortality assessment

All patients were managed in accordance with sepsis guidelines (10). Daily surveillance was performed until patients died in the hospital or were discharged. During surveillance, patients' survival status was documented to assess the 28-day mortality. Accumulating mortality was calculated from the day of admission to the day of death in the hospital or last visit.

### Statistical analysis

SPSS 22.0 (IBM, USA) was used for statistical analysis, and GraphPad Prism 7.01 (GraphPad Software, USA) was used for figure plotting. Data are reported as means±SD, or count (percentage). Comparison between groups was determined by the unpaired *t*-test. Correlation analysis between variables was determined by Pearson's test. Receiver operating characteristic (ROC) curves and the area under the curve (AUC) with 95% confidence interval (CI) were used to assess the performance of variables in predicting the susceptibility to sepsis and the 28-day mortality risk. Based on the ROC analysis of SIRT1 in predicting susceptibility to sepsis, the best statistical cut-off value (maximum sum of sensitivity and specificity) was calculated. The sensitivity, specificity, false positive rate (FPR), and false negative rate (FNR) at the best cut-off point were calculated as well. Accumulating mortality was displayed using the Kaplan-Meier method, and the mortality difference between two groups was determined by the log-rank test. A P value <0.05 was considered as statistically significant.

## Results

### Clinical characteristics of septic patient survivors and non-survivors

In the total sepsis patients, the mean age was 53.7±10.9 years, and there were 108 (60.0%) males/72 (40.0%) females (Table 1). The mean BMI of sepsis patients was 22.4±3.5 kg/m^2^. Regarding complications, 25 (13.9%), 68 (37.8%), 22 (12.2%), and 39 (21.7%) sepsis patients had COPD, cardiomyopathy, chronic kidney failure, and cirrhosis, respectively. As to primary infection site, 65 (36.1%), 40 (22.2%), 33 (18.3%), 20 (11.1%), 12 (6.7%), and 10 (5.6%) sepsis patients presented with abdominal infection, respiratory infection, skin and soft tissue infection, blood stream infection, central nervous system (CNS) infection, and other infections, respectively. In addition, the mean APACHE II score was 13.8±6.3 and the mean SOFA score was 6.3±2.8. Detailed information about other clinical characteristics of sepsis patients are shown in Table 1.

**Table 1:** Demographic and clinical characteristics of sepsis survivors and non-survivors.

Items	Total sepsis patients (n=180)	Survivors (n=129)	Non-survivors (n=51)	P value
Age (years)	53.7±10.9	53.8±11.0	53.5±10.6	0.870
Gender				0.031
Male	108 (60.0)	71 (55.0)	37 (72.5)	
Female	72 (40.0)	58 (45.0)	14 (27.5)	
BMI (kg/m^2^)	22.4±3.5	22.2±3.4	23.1±3.8	0.131
Smoking	61 (33.9)	42 (32.6)	19 (37.3)	0.549
COPD	25 (13.9)	21 (16.2)	4 (7.8)	0.140
Cardiomyopathy	68 (37.8)	54 (41.9)	14 (27.5)	0.072
Chronic kidney failure	22 (12.2)	16 (12.4)	6 (11.8)	0.906
Cirrhosis	39 (21.7)	31 (24.0)	8 (15.7)	0.221
Primary infection site				0.008
Abdominal infection	65 (36.1)	47 (36.4)	18 (35.3)	
Respiratory infection	40 (22.2)	33 (25.6)	7 (13.7)	
Skin and soft tissue infection	33 (18.3)	16 (12.4)	17 (33.3)	
Blood stream infection	20 (11.1)	18 (14.0)	2 (3.9)	
CNS infection	12 (6.7)	7 (5.4)	5 (9.8)	
Other infections	10 (5.6)	8 (6.2)	2 (3.9)	
Primary organism				
Gram-negative bacteria	104 (57.8)	77 (59.7)	27 (52.9)	0.409
Gram-positive bacteria	33 (18.3)	23 (17.8)	10 (19.6)	0.781
Anaerobes	19 (10.6)	8 (6.2)	11 (21.6)	0.002
Fungus	12 (6.7)	8 (6.2)	4 (7.8)	0.691
Mycoplasmas	6 (3.3)	3 (2.3)	3 (5.9)	0.231
Total culture negative	35 (19.4)	26 (20.2)	9 (17.6)	0.702
Biochemical indexes				
Scr (mg/dL)	2.0±1.4	1.8±1.4	2.5±1.1	0.001
Albumin (g/L)	29.8±10.4	30.7±10.7	27.5±9.4	0.066
WBC (10^9^/L)	18.2±10.6	17.1±10.9	20.8±9.5	0.038
CRP (mg/L)	107.6±83.4	89.7±72.3	152.8±92.7	<0.001
Disease severity score				
APACHE II score	13.8±6.3	12.1±5.7	17.8±6.1	<0.001
SOFA score	6.3±2.8	5.4±2.4	8.4±2.7	<0.001

Data are reported as number and percentage or mean±SD. BMI: body mass index; COPD: chronic obstructive pulmonary disease; CNS: central nervous system; Scr: serum creatinine; WBC: white blood cells; CRP: C-reactive protein; APACHE II: acute physiology and chronic health evaluation II; SOFA: sequential organ failure assessment. *t*-test or chi-squared test was used for statistical analyses.

The clinical characteristics were compared between septic survivors and non-survivors, which showed that non-survivors had significantly more female cases, less cases with primary infection site, more cases with anaerobes, higher Scr, WBC, CRP, APACHE II score, and SOFA score (all P<0.05) compared with survivors, while no difference was found in age, BMI, smoking, COPD, cardiomyopathy, chronic kidney failure, cirrhosis, gram-negative bacteria, gram-positive bacteria, fungus, mycoplasmas, total negative culture, or albumin (all P>0.05) between non-survivors and survivors (Table 1).

### SIRT1 for distinguishing sepsis patients from HCs

**Figure 1:**
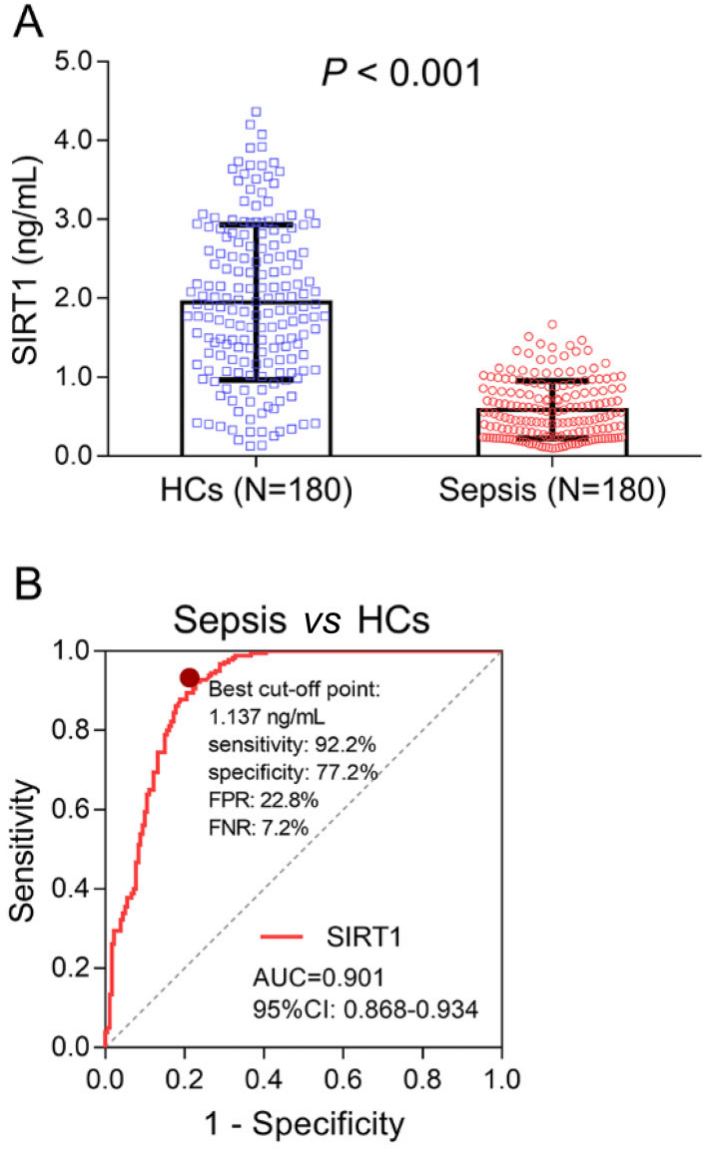
Comparison of sirtuin 1 (SIRT1) expression between healthy controls (HCs) and sepsis patients (**A**). Data are reported as means±SD (*t*-test). The performance of SIRT1 in differentiating sepsis patients from HCs (**B**).

The mean level of SIRT1 was 1.947±0.983 ng/mL in HCs and 0.587±0.365 ng/mL in sepsis patients, and comparison analysis showed that SIRT1 was significantly lower in sepsis patients compared with HCs (P<0.001) (Figure 1A). The ROC curve showed that SIRT1 had a good value in differentiating sepsis patients from HCs (AUC: 0.901, 95%CI: 0.868-0.934), with a sensitivity of 92.2%, a specificity of 77.2%, a FPR of 22.8% and a FNR of 7.2% at the best cut-off point with SIRT1 being 1.137 ng/mL (the value in which sensitivity plus specificity was the largest) (Figure 1B).

### Correlation of SIRT1 with primary infection site and primary organism in sepsis patients

**Figure 2:**
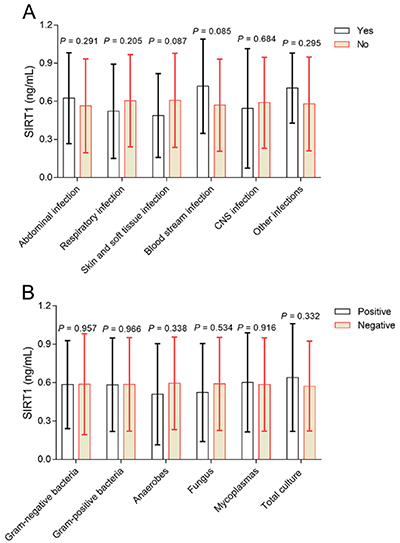
Comparisons of sirtuin 1 (SIRT1) expression between sepsis patients with (yes) and without (no) several infections (**A**), and between sepsis patients with (positive) and without (negative) bacterial total culture (**B**). Data are reported as means±SD (*t*-test). CNS: central nervous system.

No correlation of SIRT1 with abdominal infection, respiratory infection, skin and soft tissue infection, blood stream infection, CNS infection or other infections (all P>0.05) was shown in sepsis patients (Figure 2A). Furthermore, SIRT1 did not correlate with gram-negative bacteria, gram-positive bacteria, anaerobes, fungus, mycoplasmas, or total culture (all P>0.05) in sepsis patients (Figure 2B).

### Correlation of SIRT1 with biochemical indexes, SOFA score, and APACHE II score in sepsis patients

**Figure 3:**
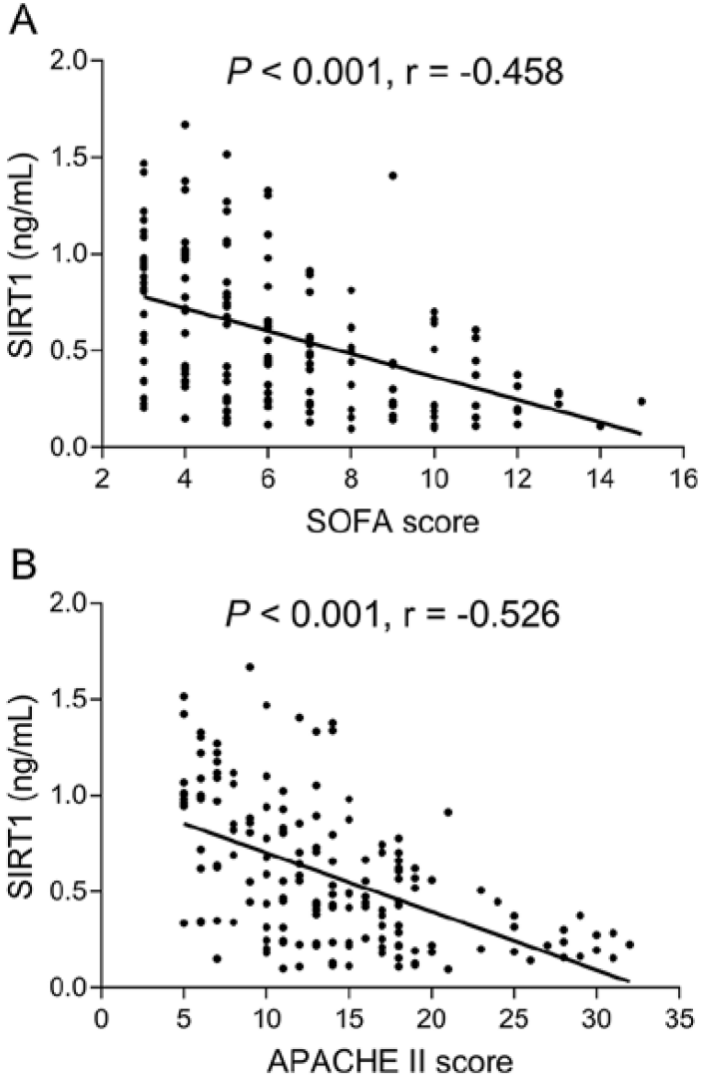
Correlation of sirtuin 1 (SIRT1) with SOFA (**A**) and APACHE II scores (**B**) in sepsis patients. SIRT1: Sirtuin 1; SOFA: sequential organ failure assessment; APACHE II: acute physiology and chronic health evaluation II. Pearson's correlation test was used for statistical analyses.

SIRT1 negatively correlated with Scr (r=-0.236), WBC (r=-0.202), and CRP (r=-0.405) and positively correlated with albumin (r=0.416) in sepsis patients (Table 2). More importantly, SIRT1 negatively correlated with SOFA score (r=-0.458) (Figure 3A) and APACHE II score (r=-0.526) (all P<0.05) (Figure 3B) in sepsis patients.

**Table 2:** Correlation of sirtuin 1 (SIRT1) with biochemical indexes.

Biochemical indexes	SIRT1	
	r	P value
Scr	-0.236	0.001
Albumin	0.416	<0.001
WBC	-0.202	0.007
CRP	-0.405	<0.001

Pearson's correlation test was used for statistical analyses. Scr: serum creatinine; WBC: white blood cells; CRP: C-reactive protein.

### SIRT1 for predicting 28-day mortality risk in sepsis patients

**Figure 4:**
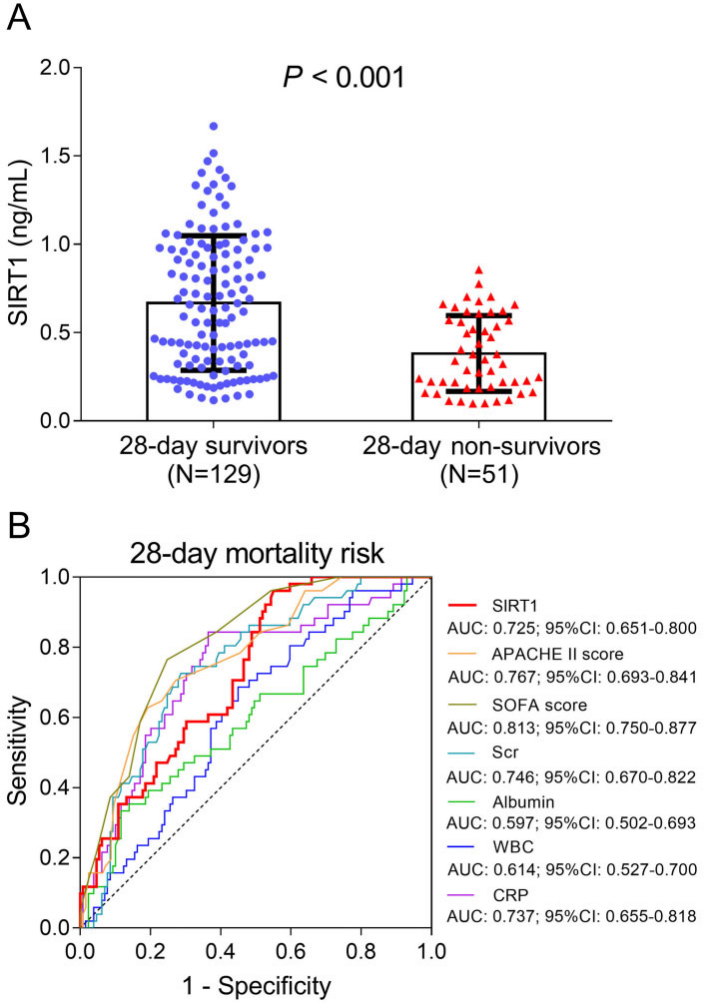
Comparison of sirtuin 1 (SIRT1) between 28-day survivors and 28-day non-survivors (**A**). The performances of SIRT1, APACHE II score, SOFA score, serum creatinine (Scr), albumin, white blood cells (WBC), and C-reactive protein (CRP) in predicting 28-day mortality risk in sepsis patients (**B**). Data are reported as means±SD (*t*-test) in panel A. APACHE II: acute physiology and chronic health evaluation II; SOFA: sequential organ failure assessment. Pearson's correlation test was used for statistical analyses.

**Figure 5:**
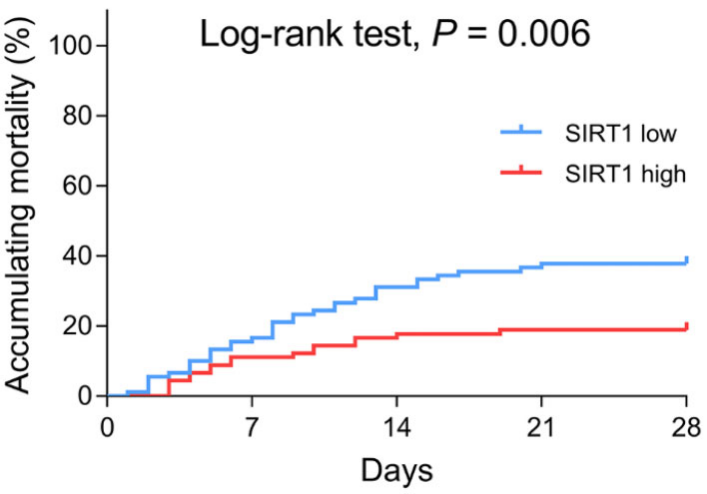
SIRT1 correlated with accumulating mortality in sepsis patients. Comparison of accumulating mortality between sirtuin 1 (SIRT1) low expression patients and SIRT1 high expression patients.

Based on the survival data, sepsis patients were divided into 28-day survivors and 28-day non-survivors. SIRT1 was decreased in 28-day non-survivors compared with 28-day survivors (P<0.001) (Figure 4A). The ROC curves showed that SIRT1 predicted 28-day mortality risk in sepsis patients, and its predictive value was not inferior to Scr, albumin, WBC, and CRP, while less than SOFA score and APACHE II score (Figure 4B). Furthermore, we divided sepsis patients into SIRT1 high group and SIRT1 low group based on their median SIRT1 level, and observed that the accumulating mortality was attenuated in sepsis patients with SIRT1 high expression compared to sepsis patients with SIRT1 low expression (P*=*0.006) (Figure 5).

## Discussion

In the present study, we found that: 1) SIRT1 was reduced in sepsis patients compared with HCs, and the ROC curve showed that SIRT1 distinguished sepsis patients from HCs; 2) SIRT1 negatively correlated with Scr, WBC, CRP, SOFA score, and APACHE II score, whereas it positively correlated with albumin in sepsis patients; and 3) SIRT1 negatively correlated with 28-day mortality, and ROC curve showed that SIRT1 predicted 28-day mortality in sepsis patients.

SIRT1, an NAD^+^-dependent class III histone deacetylase, is mainly localized in the nucleus, and it modulates the immune and inflammatory responses of sepsis to control inflammatory injury in multiple organs, and ultimately protect against multiple organ dysfunction (7,12,13). For instance, Chen et al. demonstrated that, in lipopolysaccharide-tolerant THP-1 cells of a sepsis model, resveratrol, a potential SIRT1 activator, stimulates SIRT1 activity and impedes the transcription of tumor necrosis factor-α (TNF-α) via the deacetylation of H4K16, which decrease inflammation (12). Khader et al. (13) showed that SRT1720, a SIRT1 activator, alleviates multiple organ injury via attenuating proinflammatory cytokines (e.g., interleukin (IL)-1β and IL-6) and reducing the activation of inflammasome (e.g., nucleotide oligomerization domain-like receptor protein 3 and IL-18), in a mouse model of sepsis.

Based on the effect of SIRT1 on attenuating inflammation and protecting against multiple organ dysfunction, a hypothesis was proposed that SIRT1 might be related to the occurrence and development of sepsis. In the present study, we found that SIRT1 was lower in sepsis patients than that in HCs, and it could distinguish sepsis patients from HCs. The possible explanations might be that: 1) SIRT1 probably attenuated the expression of pro-inflammatory cytokines, chemokines, and adhesion molecules in immune/endothelial cells through deacetylation and deactivation of inflammation-related factors such as NFκB p65, which inhibited inflammation in sepsis, thereby, SIRT1 was reduced in sepsis patients compared with HCs (7,14); 2) SIRT1 possibly reduced oxidative stress and repressed cell apoptosis in vital organs (e.g., lung, liver, and kidney) via downregulating various targets such as acetylated superoxide dismutase 2 (SOD2), and acetylated p53, which protected vital organs from injury, thereby, SIRT1 was lower in sepsis patients than in HCs (15,16).

Furthermore, the present study assessed the correlation of SIRT1 with inflammation and disease severity in sepsis patients. We found that SIRT1 was associated with ameliorated inflammation and disease severity (reflected by the correlation of SIRT1 with Scr, WBC, CRP, SOFA score, APACHE II score, and albumin) in sepsis patients. The findings could be explained by: 1) SIRT1 probably suppressed various downstream targets (such as inflammatory-related cytokines, nuclear factor kappa-light-chain-enhancer of activated B cells (NF-κB), high mobility group protein box 1, and TNF-α) to exert anti-inflammatory effects and protect the body from excessive inflammation; thereby, SIRTI was associated with decreased inflammation in sepsis patients (9,12,17); and 2) SIRT1 possibly downregulated the expression of pro-apoptotic proteins (e.g., acetylated p53 and Bax) and decreased oxidative stress-related factors (e.g., SOD2 and Forkhead box protein O1), which attenuated oxidative stress and cell apoptosis in vital organs such as lung, liver, and kidney, thereby, protecting vital organs from damage in sepsis; thus, SIRIT1 was associated with exacerbated disease severity in sepsis patients (15,16).

Considering our findings that SIRT1 was negatively associated with inflammation and disease severity in sepsis patients, it was speculated that SIRT1 might be related to improved prognosis as well. In the present study, we disclosed that SIRT1 was decreased in 28-day non-survivors compared with 28-day survivors, and it could predict 28-day mortality risk in sepsis patients by the ROC curve. Furthermore, Kaplan-Meier analyses exhibited that SIRT1 high was associated with lower accumulating mortality in sepsis patients. The following explanations have been proposed: 1) SIRT1 might suppress the inflammation-related signaling, attenuate oxidative stress, and repress cell apoptosis in vital organs via its downstream pathways such as NF-κB, which contributed to alleviated disease severity and lower 28-day mortality in sepsis patients (9,12,); and 2) according to our results, SIRT1 was negatively correlated with APACHE II score (reflecting disease severity) and SOFA score (reflecting the extent of organ failure and dysfunction), which indicated that SIRTI was linked to less organ injury and lower disease severity, thereby, SIRT1 was associated with decreased mortality risk in sepsis patients.

APACHE II and SOFA scores are commonly used prognostic scoring systems in sepsis patients, while they are complex (requiring multiple measurements) and time-consuming. Thus, it was necessary to explore novel and rapidly measurable prognostic biomarkers in sepsis patients (18,19). As for SIRT1, it is a single index measurement, which allowed rapid detection for indicating the prognosis in sepsis patients. In our study, although SIRT1 exhibited a slightly inferior predictive value for 28-day mortality risk compared with APACHE II score and SOFA score, it still had a clinical significance as a rapidly measurable prognostic biomarker in sepsis patients in the clinical setting.

The present study was the first to evaluate the clinical value of SIRT1 for the progression and prognosis of sepsis, however, several limitations should be noted when interpreting the findings. First, the sample size was relatively small, which might impact the statistical power of the findings. Second, SIRT1 level of sepsis patients was only detected before antibiotic therapy and the variation of SIRT1 during the antibiotic therapy was not considered. Therefore, the detection of SIRT1 at different time points during the antibiotic therapy should be assessed in the future.

To conclude, SIRT1 distinguished sepsis patients from HCs, and it correlated with lower disease severity and better prognosis in sepsis patients, which offers new perspectives for facilitating management strategies and survival surveillance of sepsis in clinical practice.
